# Combining Sodium–Glucose Co‐Transporter‐2 Inhibitor Plus Glucagon‐Like Peptide‐1 Receptor Agonist for Glucose‐Lowering in Type 2 Diabetes: Effects of Drug Initiation Sequence on Kidney Function in Real‐World Clinical Practice (CombiKid Study)

**DOI:** 10.1111/dom.70946

**Published:** 2026-06-16

**Authors:** William Hinton, Mark Joy, Anna K. Forbes, Martin B. Whyte, José M. Ordóñez‐Mena, Xuejuan Fan, Filipa Ferreira, Bernardo Meza‐Torres, Neil Munro, Simon de Lusignan, David C. Wheeler, Michael D. Feher

**Affiliations:** ^1^ Nuffield Department of Primary Care Health Sciences University of Oxford Oxford UK; ^2^ Department of Clinical and Experimental Medicine University of Surrey Guildford UK; ^3^ Renal Services, Epsom and St. Helier University Hospitals NHS Trust London UK; ^4^ Royal College of General Practitioners (RCGP) Research and Surveillance Centre (RSC) London UK; ^5^ Centre for Kidney and Bladder Health University College London London UK

**Keywords:** cohort studies, glucagon‐like peptide‐1 receptor agonists, kidney function tests, sodium‐glucose transporter 2 inhibitors, type 2 diabetes mellitus

## Abstract

**Aims:**

Sodium–glucose co‐transporter 2 inhibitors (SGLT2is) and glucagon‐like peptide‐1 receptor agonists (GLP‐1 RAs) have separately shown renoprotective effects in clinical trials in people with type 2 diabetes. It is unclear whether combining these agents produces incremental kidney outcome benefits.

**Materials and Methods:**

Retrospective cohort study with a prevalent new‐user design using pseudonymised data from the Oxford‐Royal College of General Practitioners Research and Surveillance Centre primary care sentinel network. We extracted data for two cohorts prescribed SGLT2is and/or GLP‐1 RAs between January 2013 and December 2021. We 1:1 propensity score matched SGLT2i plus GLP‐1 RA combination users with monotherapy (SGLT2i or GLP‐1RA) users. Multivariable linear regression analyses estimated adjusted mean differences in absolute change in estimated glomerular filtration rate (eGFR) (baseline to 1‐ and 2‐years follow‐up) between combination and monotherapy.

**Results:**

Across the matched combination and SGLT2i monotherapy groups (*N* = 14 774), mean decline in eGFR, baseline to 1‐year, was −4.5 mL/min/1.73 m^2^ and 2‐years, −5.0 mL/min/1.73 m^2^, with no difference when comparing combination and SGLT2i monotherapy. Across the matched combination and GLP‐1 RA monotherapy groups (*N* = 14 154), mean decline in eGFR, baseline to 1‐year, was −5.4 mL/min/1.73 m^2^ and 2‐years, −6.1 mL/min/1.73 m^2^, but combination therapy was associated with a smaller eGFR reduction compared with GLP‐1 RA monotherapy (difference in eGFR at 1‐year: [mean, 95% confidence interval, CI] 2.2, 1.7 to 2.8, *p* < 0.001; and at 2‐years: 2.1, 1.4–2.7, *p* < 0.001).

**Conclusions:**

In real‐world clinical practice, the combination of SGLT2i and GLP‐1 RA may be more effective for preserving kidney function than GLP‐1 RA monotherapy. This effect was not seen with combination versus SGLT2i monotherapy.

## 1 Introduction

Guidelines for the management of type 2 diabetes recommend that sodium–glucose co‐transporter 2 inhibitors (SGLT2is) and glucagon‐like peptide‐1 receptor agonists (GLP‐1 RAs) may be used in combination for glucose control [1, 2]. Additionally, both drug classes have separately shown kidney benefits [3, 4, 5, 6, 7, 8]. However, it is unclear whether there might be an additional kidney protective benefit of prescribing an SGLT2i in a patient already receiving a GLP‐1 RA or vice versa.

Most data for kidney effects with these drug combinations come from the few post hoc or secondary analyses of cardiovascular outcome trials of high‐risk populations. These are limited in both duration and the range of kidney‐specific endpoints [9, 10, 11, 12]. Only a few observational studies have evaluated kidney endpoints with these drug combinations in real‐world clinical practice in lower risk type 2 diabetes populations [13, 14, 15, 16, 17], but these studies lack data on biochemical measurements of kidney function. As the use of SGLT2is and GLP‐1 RAs increases, understanding their impact on kidney outcomes, both when used alone and in combination, is important.

We explored the effects of combining SGLT2is and GLP‐1 RAs on biochemical measures of kidney function in people with type 2 diabetes in real‐world clinical practice. The primary objective was to investigate differences in change in estimated glomerular filtration rate (eGFR) over time between matched users of SGLT2i plus GLP‐1 RA combination versus monotherapy users (either SGLT2i or GLP‐1 RA) from a baseline of transitioning to combination therapy to 1‐ and 2‐years follow‐up. Secondary objectives were to assess differences between combination and monotherapy users in urine albumin‐creatinine ratio (UACR), glycated haemoglobin (HbA1c), and body mass index (BMI), at the same time points.

## 2 Materials and Methods

### 2.1 Study Design and Data Source

We performed a retrospective cohort study with an active comparator, prevalent new‐user design, using routinely collected data from computerised medical records (CMRs) of patients registered with primary care practices in the Oxford‐Royal College of General Practitioners (RCGP) Research and Surveillance Centre (RSC) network.

The RSC comprises over 1900 volunteer practices representing over 19 million patients across England and Wales, representative of the national population [18]. At the time of data request (31 December 2022), the RSC database included 738 practices and 6 670 829 adult patients.

We analysed pseudonymised CMR data recorded using clinical codes. Clinical coding was with the Systematised Nomenclature of Medicine Clinical Terms (SNOMED CT) format [19].

The protocol for this study has previously been published [20]. Some changes to the methods of the study protocol are described (Supporting Information 1).

### 2.2 Base Cohorts

We identified patients with type 2 diabetes using a two‐step ontological‐based approach [21]. Within this cohort, we selected individuals aged ≥ 40 years at diagnosis and prescribed at least one SGLT2i (canagliflozin, dapagliflozin, empagliflozin, ertugliflozin), and/or one GLP‐1 RA (dulaglutide, exenatide, liraglutide, lixisenatide, semaglutide) between 1 January 2013 and 31 December 2021. We restricted the study population to those registered with an RSC practice for at least 12 months prior to the first prescription of a drug from either medication class to ensure that only new users of SGLT2is or GLP‐1 RAs were included in the analyses. We then assembled two separate base cohorts. The first base cohort comprised SGLT2i users that had no previous prescriptions of GLP‐1 RAs. The second base cohort included GLP‐1 RA users that had no previous prescriptions of SGLT2is.

We defined users of SGLT2 is plus GLP‐1 RA combination therapy according to prescriptions of a SGLT2i and GLP‐1 RA within 90 days of each other.

### 2.3 Time‐Based Exposure Sets

To identify appropriate monotherapy comparator drug users, we constructed exposure sets based on the timing of combination therapy initiation within each base cohort. In the first base cohort, we divided the follow‐up period (1 January 2013 –31 December 2021) into 90‐day intervals, fixed at the timepoint individuals had a first GLP‐1 RA prescription added to their existing SGLT2i regimen. A monotherapy user entered an exposure set when a new SGLT2i prescription fell within the time interval of an individual switching to combination therapy (Figure S1), ensuring comparability at similar disease stages [22]. We repeated this process for the second base cohort, which included individuals that had an SGLT2i added to their existing GLP‐1 RA regimen. We allowed for an additional year of follow‐up (up to 31 December 2022) to account for individuals that entered towards the end of the cohort entry period.

The index date (baseline) was defined according to the point at which individuals transitioned to combination therapy. Comparators in the corresponding exposure set shared the same index dates.

### 2.4 Outcomes Measures

The primary outcomes were the absolute changes in eGFR (from baseline to 1‐ and 2‐years follow‐up) between combination and monotherapy (SGLT2i or GLP‐1 RA). To calculate eGFR, we used the Chronic Kidney Disease Epidemiology Collaboration (CKD‐EPI) 2021 equation [23, 24]. Individuals were required to have a baseline eGFR measurement and at least one eGFR measurement post index date at 1‐ and/or 2‐years follow‐up.

Secondary outcomes were absolute changes in UACR, HbA1c, and BMI (from baseline to 1‐ and 2‐years follow‐up) between combination and monotherapy (SGLT2i or GLP‐1 RA).

We defined the measurements for 1‐ and 2‐years follow‐up with an allowance of 3 months (+/−) on either side.

### 2.5 Covariates

We accounted for the following variables: age, sex, ethnicity (White, Asian, Black, Mixed, Other), socioeconomic status, and BMI; history of hypertension, cardiovascular disease, heart failure, haemorrhagic stroke, neuropathy, retinopathy, valvular heart disease; use of angiotensin‐converting enzyme (ACE) inhibitors, angiotensin receptor blockers (ARBs), diuretics, beta blockers, statins, acarbose, dipeptidyl peptidase‐4 (DPP‐4) inhibitors, insulin, metformin, sulphonylureas, thiazolidinediones, non‐steroidal anti‐inflammatory drugs (NSAIDs); as well as duration of diabetes, HbA1c, eGFR, and UACR.

Age and duration of diabetes were defined at the index date (date of switching to combination therapy). Concomitant medication use was defined within a 90‐day interval of the index date. The eGFR, UACR, HbA1c, and BMI were defined according to latest measurements before the index date. Comorbidities were those ever recorded before the index date. Socioeconomic status was determined using index of multiple deprivation (IMD) scores [25]. These were converted into quintiles, ranging from 1 (most deprived) to 5 (least deprived).

### 2.6 Propensity Score Matching

Propensity score matching was performed separately for each base cohort. Within each exposure set, we estimated propensity scores according to the likelihood of switching (from SGLT2i or GLP‐1 RA monotherapy) to SGLT2i plus GLP‐1 RA combination therapy, using multivariable logistic regression with the above‐described covariates.

We then matched the combination new users to the monotherapy new users in chronological order within the same exposure sets, according to propensity score. This was on a 1:1 basis using nearest neighbour matching with replacement and Mahalanobis distance. An individual that was matched as a monotherapy user but later switched to combination therapy was a prevalent new user and could be included again for matching against another monotherapy user.

### 2.7 Statistical Analyses

Baseline characteristics are reported using medians (interquartile range [IQR]) for continuous variables and counts (with percentages) for categorical variables. We used standardised differences to assess balance pre‐ and post‐matching, with meaningful differences considered ≥ 0.1.

This was an analysis of complete matched cases using an intention‐to‐treat design. For both the primary and secondary outcomes, we estimated the mean differences in absolute changes (from index date to 1‐ and 2‐years follow‐up) between combination users and monotherapy users (SGLT2is or GLP‐1 RAs) using linear regression. Analyses were performed unadjusted and adjusted for all baseline covariates.

All data analyses were undertaken using R Statistical Software, version 4.5.1 (2025‐06‐13). R packages used are provided (Supporting Information 1).

### 2.8 Subgroup Analyses

We stratified the cohorts according to the presence of CKD at baseline, defined as the latest eGFR < 60 mL/min/1.73 m^2^ prior to the index date. We also stratified the cohorts based on whether UACR was < 3 or ≥ 3 mg/mmol at baseline [26], and whether HbA1c was < 53 mmol/mol, between ≥ 53 and < 64 mmol/mol, or ≥ 64 mmol/mol. We then re‐ran the primary analysis according to concomitant use of renin‐angiotensin system (RAS) inhibitors (ACE inhibitors or ARBs). Concomitant medication use was defined as 90 days within the index date.

### 2.9 Sensitivity Analyses

For sensitivity analysis, we only included individuals that transitioned to combination therapy up to 31 December 2019 to account for reduced access to healthcare services during the COVID‐19 pandemic [27]. Separately, we accounted for missing data by assigning these to an ‘unknown’ category in each affected variable before propensity score matching.

### 2.10 Ethics Statement

Ethical approval was granted by the Medical Sciences Interdivisional Research Ethics Committee, University of Oxford (reference number: R76885/RE004). Patient data were pseudonymised at the time of extraction and were retrieved from individuals who did not dissent for their data to be used for purposes of secondary research.

## 3 Results

### 3.1 SGLT2i Plus GLP‐1 RA Combination Users Compared With SGLT2i Monotherapy Users

We identified 72 299 individuals prescribed SGLT2is between 1 January 2013 and 31 December 2021 (Figure S2). Of these, 60 647 individuals were identified as new users of SGLT2is. Before propensity score matching, higher proportions of SGLT2i plus GLP‐1 RA combination users were of white ethnicity, had higher HbA1c and BMI, and were more likely to have retinopathy and be prescribed statins, NSAIDs, and other glucose‐lowering medications than SGLT2i monotherapy users (Table S1). However, they were slightly younger, less likely to be male, and have heart failure.

After propensity score matching, there were 7387 combination users and an equal number of SGLT2i monotherapy users (Figure S2). The groups were well balanced across all measured baseline covariates, except for HbA1c and BMI (Table 1 and Figure S3). The median duration of follow‐up of the matched cohort was 2.7 years (IQR 1.5 to 4.1) (Figure S4).

**TABLE 1:** Baseline characteristics of SGLT2i plus GLP‐1 RA combination and SGLT2i monotherapy new users post matching.

Characteristics	SGLT2i and GLP‐1 RA combination cohort (*N* = 7387)	SGLT2i monotherapy cohort (*N* = 7387)	Std. diff.
Median (IQR) age, years	62.2 (56.4–68.2)	61.5 (55.5–67.9)	0.085
Male	4406 (59.6)	4134 (56.0)	0.075
Ethnicity			
White	6716 (90.9)	6699 (90.7)	0.007
Asian	394 (5.3)	411 (5.6)	0.013
Black	156 (2.1)	156 (2.1)	0.000
Mixed	52 (0.7)	52 (0.7)	0.000
Other	69 (0.9)	69 (0.9)	0.000
IMD quintile			
5 (least deprived)	1261 (17.1)	1264 (17.1)	0.000
4	1450 (19.6)	1458 (19.7)	0.003
3	1443 (19.5)	1447 (19.6)	0.003
2	1518 (20.5)	1528 (20.7)	0.005
1 (most deprived)	1715 (23.2)	1690 (22.9)	0.007
Median (IQR) duration of diabetes, years	9.9 (6.4–13.6)	9.7 (6.1–13.9)	0.017
Duration of diabetes categories			
< 1 year	92 (1.2)	51 (0.7)	0.052
1–5 years	1069 (14.5)	1245 (16.9)	0.066
5–10 years	2603 (35.2)	2561 (34.7)	0.010
≥ 10 years	3623 (49.0)	3530 (47.8)	0.024
Median (IQR) BMI, kg/m^2^	32.9 (29.7–36.8)	34.3 (30.9–38.7)	0.263
BMI categories			
Underweight	< 5 (0.0)	6 (0.1)	0.045
Normal weight	234 (3.2)	151 (2.0)	0.075
Overweight	1727 (23.4)	1230 (16.7)	0.168
Obese	5425 (73.4)	6000 (81.2)	0.187
Median (IQR) HbA1c, mmol/mol	66.0 (57.0–77.0)	72.0 (63.0–84.0)	0.372
Median (IQR) HbA1c, %	8.2 (7.4–9.2)	8.7 (7.9–9.8)	0.270
HbA1c categories			
< 53 mmol/mol (< 7%)	1020 (13.8)	586 (7.9)	0.191
53–63 mmol/mol (7%–8%)	2172 (29.4)	1342 (18.2)	0.265
64–74 mmol/mol (8%–9%)	2028 (27.5)	2157 (29.2)	0.038
75–85 mmol/mol (9%–10%)	1136 (15.4)	1627 (22.0)	0.170
≥ 86 mmol/mol (10%)	1031 (14.0)	1675 (22.7)	0.226
Median (IQR) UACR, mg/mmol	1.2 (0.6–2.5)	1.3 (0.6–2.9)	0.053
UACR categories			
< 3 mg/mmol	5803 (78.6)	5585 (75.6)	0.071
3–30 mg/mmol	99 (1.3)	195 (2.6)	0.094
> 30 mg/mmol	1485 (20.1)	1607 (21.8)	0.042
Median (IQR) eGFR, mL/min/1.73 m^2^	91.5 (81.3–100.0)	91.9 (80.6–101.1)	0.009
eGFR categories			
< 60 mL/min/1.73 m^2^	51 (0.7)	105 (1.4)	0.069
≥ 60 mL/min/1.73 m^2^	7336 (99.3)	7282 (98.6)	0.069
Comorbidities			
Hypertension	5594 (75.7)	5452 (73.8)	0.044
CVD	1666 (22.6)	1783 (24.1)	0.037
Heart failure	514 (7.0)	531 (7.2)	0.009
Haemorrhagic stroke	69 (0.9)	69 (0.9)	< 0.001
Neuropathy	579 (7.8)	591 (8.0)	0.006
Retinopathy	3159 (42.8)	3257 (44.1)	0.027
Valvular heart disease	296 (4.0)	299 (4.0)	0.002
Medications			
ACE inhibitor	1408 (19.1)	1467 (19.9)	0.020
ARB	3634 (49.2)	3574 (48.4)	0.016
NSAID	1049 (14.2)	1169 (15.8)	0.045
Acarbose	13 (0.2)	13 (0.2)	< 0.001
Beta blocker	1687 (22.8)	1829 (24.8)	0.045
Diuretic	1578 (21.4)	1675 (22.7)	0.032
Metformin	6492 (87.9)	6399 (86.6)	0.038
Statin	6260 (84.7)	6136 (83.1)	0.046
Sulphonylurea	2873 (38.9)	3087 (41.8)	0.059
DPP‐4 inhibitor	2761 (37.4)	3063 (41.5)	0.084
Insulin	1060 (14.3)	1165 (15.8)	0.040

Abbreviations: ACE = angiotensin‐converting enzyme; ARB = angiotensin receptor blockers; BMI = body mass index; CVD = cardiovascular disease; DPP‐4 = dipeptidyl peptidase 4; eGFR = estimated glomerular filtration rate; HbA1c = glycated haemoglobin; IMD = index of multiple deprivation; IQR = interquartile range; NSAID = non‐steroidal anti‐inflammatory drug; Std. diff. = standardised difference; UACR = urine albumin to creatinine ratio.

Compared with baseline, eGFR declined in both combination and SGLT2i monotherapy users at 1‐year (−4.4 and −4.7 mL/min/1.73 m^2^, respectively) and 2‐years (−5.1 and −5.0 mL/min/1.73 m^2^, respectively) follow‐up. There was no difference in eGFR change between combination and SGLT2i monotherapy use at both timepoints (change in eGFR at 1‐year: [adjusted mean difference, 95% confidence interval, CI] 0.4, −0.14 to 0.88, *p* = 0.158; and at 2‐years: −0.3, −0.96 to 0.31, *p* = 0.310).

There was also no difference between combination and SGLT2i monotherapy for changes in UACR at 1‐ and 2‐years follow‐up (Table 2). Combination therapy was associated with a larger reduction in HbA1c compared with SGLT2 monotherapy users at both timepoints. But there was no difference in the changes at 1‐ and 2‐years for BMI between combination therapy and SGLT2i monotherapy.

**TABLE 2:** Crude and adjusted mean differences in primary and secondary outcomes for the comparison between SGLT2i plus GLP‐1 RA combination therapy and SGLT2i monotherapy.

Outcome	No. with a measurement	Mean outcome	Crude mean difference (95% CI)	Adjusted mean difference (95% CI)^a^
Exposure group				
eGFR—1 year (mL/min/1.73 m^2^)				
SGLT2i + GLP‐1 RA	4851	−4.4	0.4 (−0.25 to 1.01)	0.4 (−0.14 to 0.88)
SGLT2i	4559	−4.7	0 (Reference)	0 (Reference)
eGFR—2 years (mL/min/1.73 m^2^)				
SGLT2i + GLP‐1 RA	3314	−5.1	−0.2 (−0.43 to 0.67)	−0.3 (−0.96 to 0.31)
SGLT2i	3239	−5.0	0 (Reference)	0 (Reference)
UACR—1 year (mg/mmol)				
SGLT2i + GLP‐1 RA	2094	3.4	1.8 (−0.80 to 4.44)	1.5 (−1.22 to 4.15)
SGLT2i	1959	1.6	0 (Reference)	0 (Reference)
UACR—2 years (mg/mmol)				
SGLT2i + GLP‐1 RA	1435	1.3	−1.7 (−3.42 to 0.05)	−1.5 (−3.21 to 0.25)
SGLT2i	1409	3.0	0 (Reference)	0 (Reference)
HbA1c—1 year (mmol/mol)				
SGLT2i + GLP‐1 RA	4856	−6.6	−7.6 (−8.29 to −6.82)	−3.9 (−4.56 to −3.24)
SGLT2i	4248	0.9	0 (Reference)	0 (Reference)
HbA1c—2 years (mmol/mol)				
SGLT2i + GLP‐1 RA	3349	−5.4	−6.0 (−6.92 to −5.09)	−2.0 (−2.82 to −1.18)
SGLT2i	3145	0.6	0 (Reference)	0 (Reference)
BMI—1 year (kg/m^2^)				
SGLT2i + GLP‐1 RA	3993	−1.0	−0.2 (−0.37 to −0.12)	−0.0 (−0.16 to 0.09)
SGLT2i	3357	−0.8	0 (Reference)	0 (Reference)
BMI—2 years (kg/m^2^)				
SGLT2i + GLP‐1 RA	2646	−1.0	−0.0 (−0.22 to 0.13)	0.2 (−0.01 to 0.35)
SGLT2i	2445	−1.0	0 (Reference)	0 (Reference)

Abbreviations: BMI = body mass index; CI = confidence interval; eGFR = estimated glomerular filtration rate; HbA1c = glycated haemoglobin; UACR = urine albumin to creatinine ratio.

^a^ Further adjusted for age, sex, ethnicity, socioeconomic status, BMI, hypertension, cardiovascular disease, heart failure, haemorrhagic stroke, neuropathy, retinopathy, valvular heart disease, ACE inhibitors, ARB, diuretics, beta blockers, statins, acarbose, DPP‐4 inhibitors, insulin, metformin, sulphonylureas, thiazolidinediones, NSAID, duration of diabetes, HbA1c, eGFR, and UACR.

#### 3.1.1 Subgroup Analyses

The majority of the cohort (98.9%) had a baseline eGFR ≥ 60 mL/min/1.73 m^2^. Due to only *n* = 156 having an eGFR < 60 mL/min/1.73 m^2^, subgroup analysis based on stage of CKD was not possible.

After stratification by UACR, HbA1c, or RAS inhibitor use, no differences in eGFR changes were detected between combination and SGLT2i monotherapy users (Tables S2–S4).

#### 3.1.2 Sensitivity Analyses

Sensitivity analyses (individuals initiating combination therapy before 31 December 2019, and accounting for missing data in matching) were largely consistent with the main findings, showing no difference in eGFR change at 1‐year but a larger decline at 2‐years in one analysis for combination therapy compared with SGLT2i monotherapy (Tables S5 and S6).

### 3.2 SGLT2i Plus GLP‐1 RA Combination Users Compared With GLP‐1 RA Monotherapy Users

We identified 24 909 individuals prescribed GLP‐1 RAs between 1 January 2013 and 31 December 2021 (Figure S5). Of these, 20 184 individuals were identified as new users of GLP‐1 RAs. Before propensity score matching, SGLT2i plus GLP‐1 RA combination users had a longer duration of diabetes, higher eGFR, and were more likely to be prescribed metformin, statins, and DPP‐4 inhibitors than GLP‐1 RA monotherapy users (Table S7). However, they were slightly younger, had a lower BMI and UACR, were less likely to have heart failure, and less likely to be prescribed diuretics and insulin.

After 1:1 propensity score matching, there were 7077 pairs of combination users and GLP‐1 RA monotherapy users (Figure S5). These groups were well balanced across all measured baseline covariates, except for HbA1c (Table 3 and Figure S6). The median duration of follow‐up of the matched cohort was 2.1 years (IQR 0.5–3.8) (Figure S7).

**TABLE 3:** Baseline characteristics of SGLT2i plus GLP‐1 RA combination and GLP‐1 RA monotherapy new users post matching.

Characteristics	SGLT2i and GLP‐1 RA combination cohort (*N* = 7077)	GLP‐1 RA monotherapy cohort (*N* = 7077)	Std. diff.
Median (IQR) age, years	61.5 (55.4–67.8)	61.6 (55.8–67.5)	0.024
Male	3942 (55.7)	4046 (57.2)	0.030
Ethnicity			
White	6407 (90.5)	6412 (90.6)	0.003
Asian	399 (5.6)	395 (5.6)	0.000
Black	152 (2.1)	152 (2.1)	0.000
Mixed	52 (0.7)	52 (0.7)	0.000
Other	67 (0.9)	66 (0.9)	0.000
IMD quintile			
5 (least deprived)	1212 (17.1)	1189 (16.8)	0.008
4	1400 (19.8)	1368 (19.3)	0.013
3	1399 (19.8)	1408 (19.9)	0.003
2	1450 (20.5)	1452 (20.5)	0.000
1 (most deprived)	1616 (22.8)	1660 (23.5)	0.017
Median (IQR) duration of diabetes, years	9.7 (6.1–13.8)	9.8 (6.5–13.6)	0.003
Duration of diabetes categories			
< 1 year	47 (0.7)	75 (1.1)	0.042
1–5 years	1181 (16.7)	1001 (14.1)	0.072
5–10 years	2474 (35.0)	2545 (36.0)	0.021
≥ 10 years	3375 (47.7)	3456 (48.8)	0.022
Median (IQR) BMI, kg/m^2^	34.3 (31.0–38.7)	34.4 (31.2–38.1)	0.045
BMI categories			
Underweight	5 (0.1)	< 5 (0.0)	0.045
Normal weight	140 (2.0)	85 (1.2)	0.064
Overweight	1174 (16.6)	1076 (15.2)	0.038
Obese	5758 (81.4)	5914 (83.6)	0.058
Median (IQR) HbA1c, mmol/mol	72.0 (63.0–84.0)	66.0 (57.0–78.0)	0.377
Median (IQR) HbA1c, %	8.7 (7.9–9.8)	8.2 (7.4–9.3)	0.263
HbA1c categories			
< 53 mmol/mol (< 7%)	545 (7.7)	1085 (15.3)	0.240
53–63 mmol/mol (7%–8%)	1253 (17.7)	1968 (27.8)	0.243
64–74 mmol/mol (8%–9%)	2130 (30.1)	1864 (26.3)	0.085
75–85 mmol/mol (9%–10%)	1537 (21.7)	1133 (16.0)	0.146
≥ 86 mmol/mol (10%)	1612 (22.8)	1027 (14.5)	0.214
Median (IQR) UACR, mg/mmol	1.3 (0.6–2.9)	1.3 (0.6–2.6)	0.050
UACR categories			
< 3 mg/mmol	5361 (75.8)	5483 (77.5)	0.040
3–30 mg/mmol	1530 (21.6)	1485 (21.0)	0.015
> 30 mg/mmol	186 (2.6)	109 (1.5)	0.078
Median (IQR) eGFR, mL/min/1.73 m^2^	91.9 (80.6–101.1)	91.9 (81.2–100.3)	0.001
eGFR categories			
< 60 mL/min/1.73 m^2^	98 (1.4)	79 (1.1)	0.027
≥ 60 mL/min/1.73 m^2^	6979 (98.6)	6998 (98.9)	0.027
Comorbidities			
Hypertension	5227 (73.9)	5354 (75.7)	0.041
CVD	1699 (24.0)	1500 (21.2)	0.067
Heart failure	506 (7.1)	451 (6.4)	0.031
Haemorrhagic stroke	65 (0.9)	65 (0.9)	< 0.001
Neuropathy	575 (8.1)	540 (7.6)	0.018
Retinopathy	3140 (44.4)	3050 (43.1)	0.026
Valvular heart disease	279 (3.9)	276 (3.9)	0.002
Medications			
ACE inhibitor	1408 (19.9)	1287 (18.2)	0.044
ARB	3408 (48.2)	3541 (50.0)	0.038
NSAID	1161 (16.4)	1014 (14.3)	0.058
Acarbose	12 (0.2)	12 (0.2)	< 0.001
Beta blocker	1730 (24.4)	1557 (22.0)	0.058
Diuretic	1597 (22.6)	1468 (20.7)	0.044
Metformin	6150 (86.9)	6303 (89.1)	0.067
Statin	5861 (82.8)	6002 (84.8)	0.054
Sulphonylurea	2979 (42.1)	2821 (39.9)	0.045
DPP‐4 inhibitor	2940 (41.5)	2626 (37.1)	0.091
Insulin	1120 (15.8)	1010 (14.3)	0.043

Abbreviations: ACE = angiotensin‐converting enzyme; ARB = angiotensin receptor blockers; BMI = body mass index; CVD = cardiovascular disease; DPP‐4 = dipeptidyl peptidase 4; eGFR = estimated glomerular filtration rate; HbA1c = glycated haemoglobin; IMD = index of multiple deprivation; IQR = interquartile range; NSAID = non‐steroidal anti‐inflammatory drug; Std. diff. = standardised difference; UACR = urine albumin to creatinine ratio.

Compared with baseline, eGFR declined in both combination and GLP‐1 RA monotherapy users at 1‐year (−4.4 and −6.4 mL/min/1.73 m^2^, respectively) and 2‐years (−5.2 and −7.1 mL/min/1.73 m^2^, respectively) follow‐up. However, combination therapy was associated with a smaller eGFR reduction compared with GLP‐1 RA monotherapy (at 1‐year: [adjusted mean difference, 95% CI] 2.2, 1.68 to 2.78, *p* < 0.001; and at 2‐years: 2.1, 1.39 to 2.73, *p* < 0.001).

For secondary outcomes, smaller increases at 2‐years follow‐up were observed in UACR ([adjusted mean difference, 95% CI] −2.4 mg/mmol, −4.44 to −0.32, *p* = 0.023) for combination therapy compared with GLP‐1 RA monotherapy. HbA1c and BMI reduced at 1‐ and 2‐years in the combination therapy group compared with the GLP‐1 RA monotherapy group (Table 4).

**TABLE 4:** Crude and adjusted mean differences in primary and secondary outcomes for the comparison between SGLT2i plus GLP‐1 RA combination therapy and GLP‐1 RA monotherapy.

Outcome	No. with a measurement	Mean outcome	Crude mean difference (95% CI)	Adjusted mean difference (95% CI)^a^
Exposure group				
eGFR—1 year (mL/min/1.73 m^2^)				
SGLT2i + GLP‐1 RA	4614	−4.4	2.0 (1.34 to 2.67)	2.2 (1.68 to 2.78)
GLP‐1 RA	4456	−6.4	0 (Reference)	0 (Reference)
eGFR—2 years (mL/min/1.73 m^2^)				
SGLT2i + GLP‐1 RA	3303	−5.2	1.9 (1.11 to 2.72)	2.1 (1.39 to 2.73)
GLP‐1 RA	3371	−7.1	0 (Reference)	0 (Reference)
UACR—1 year (mg/mmol)				
SGLT2i + GLP‐1 RA	2047	3.7	1.0 (−1.47 to 3.41)	−0.4 (−2.85 to 2.07)
GLP‐1 RA	2045	2.8	0 (Reference)	0 (Reference)
UACR—2 years (mg/mmol)				
SGLT2i + GLP‐1 RA	1447	2.1	−1.9 (−3.85 to 0.15)	−2.4 (−4.44 to −0.32)
GLP‐1 RA	1554	4.0	0 (Reference)	0 (Reference)
HbA1c—1 year (mmol/mol)				
SGLT2i + GLP‐1 RA	4991	−6.6	−7.4 (−8.09 to −6.65)	−4.0 (−4.63 to −3.35)
GLP‐1 RA	4835	0.8	0 (Reference)	0 (Reference)
HbA1c—2 years (mmol/mol)				
SGLT2i + GLP‐1 RA	3551	−5.6	−6.7 (−7.61 to −5.83)	−2.6 (−3.39 to −1.81)
GLP‐1 RA	3605	1.1	0 (Reference)	0 (Reference)
BMI—1 year (kg/m^2^)				
SGLT2i + GLP‐1 RA	3955	−1.0	−0.4 (−0.57 to −0.32)	−0.4 (−0.52 to −0.26)
GLP‐1 RA	3810	−0.5	0 (Reference)	0 (Reference)
BMI—2 years (kg/m^2^)				
SGLT2i + GLP‐1 RA	2777	−1.0	−0.3 (−0.43 to −0.08)	−0.2 (−0.39 to −0.04)
GLP‐1 RA	2813	−0.7	0 (Reference)	0 (Reference)

Abbreviations: BMI = body mass index; CI = confidence interval; eGFR = estimated glomerular filtration rate; HbA1c = glycated haemoglobin; UACR = urine albumin to creatinine ratio.

^a^ Further adjusted for age, sex, ethnicity, socioeconomic status, BMI, hypertension, cardiovascular disease, heart failure, haemorrhagic stroke, neuropathy, retinopathy, valvular heart disease, ACE inhibitors, ARB, diuretics, beta blockers, statins, acarbose, DPP‐4 inhibitors, insulin, metformin, sulphonylureas, thiazolidinediones, NSAID, duration of diabetes, HbA1c, eGFR, and UACR.

#### 3.2.1 Subgroup Analyses

Almost all individuals (98.7%) had a baseline eGFR ≥ 60 mL/min/1.73 m^2^. Only *n* = 177 individuals had eGFR < 60 mL/min/1.73 m^2^, therefore, subgroup analysis was not performed.

Subgroup analyses stratified by UACR level, HbA1c value, or RAS inhibitor use showed consistent findings, with smaller eGFR reductions in combination users compared with GLP‐1 RA monotherapy (Tables S8–S10). However, in individuals with a baseline HbA1c < 53 mmol/mol, at 2‐years follow‐up, the difference in change in eGFR between combination and GLP‐1 RA monotherapy users was not statistically significant (Table S9).

#### 3.2.2 Sensitivity Analyses

Sensitivity analyses were also consistent with the main findings; combination therapy was associated with smaller eGFR reductions at 1‐ and 2‐year follow‐up compared with GLP‐1 RA monotherapy (Tables S11 and S12).

## 4 Discussion

This is one of the first large real‐world observational studies to assess the effect of combining SGLT2i plus GLP‐1 RA therapy on biochemical markers of kidney function in a cohort with type 2 diabetes with predominantly normal baseline eGFR. Analyses showed improvements in eGFR and UACR within 2 years of combination therapy compared with GLP‐1 RA use alone. By contrast, adding a GLP‐1 RA to baseline SGLT2i treatment compared with SGLT2i monotherapy showed no effect for either of the kidney outcomes. Importantly, as the drugs were prescribed for glucose‐lowering, it is reassuring that HbA1c improved within 2 years after starting combination therapy compared with either drug class alone.

### 4.1 Established Kidney Benefits of Each Drug Class

Randomised controlled trials demonstrated that SGLT2is significantly reduce the risk of sustained decline in eGFR, progression to end‐stage kidney disease, and kidney‐related mortality [5, 6, 7], likely through mechanisms including reduced intraglomerular pressure, improved tubular‐glomerular feedback, and attenuation of renal inflammation and fibrosis [28, 29]. SGLT2is are now recommended as cornerstone therapy in diabetic kidney disease [1]. GLP‐1 RAs have similarly shown beneficial effects on composite kidney outcomes through reductions in albuminuria and slowing of renal function decline [3, 4, 8], possibly through improved glycaemic control, anti‐inflammatory effects, and reduction in blood pressure and body weight [30, 31, 32]. Although a recent meta‐analysis of trials involving GLP‐1 RAs suggested that their effects on preserving eGFR may only be modest to negligible [33].

### 4.2 Which Baseline Drug Is Important for Combination Effect?

Our study, reflecting real‐world clinical practice, suggests that adding an SGLT2i to baseline GLP‐1 RA therapy may provide greater kidney protection (than GLP‐1 RA monotherapy) within the first 2 years of switching to combination therapy. When the other combination was assessed (GLP‐1 RA added to SGLT2i) compared with SGLT2i monotherapy, there was no observed kidney benefit. Whilst we did not make direct comparisons of these different therapeutic strategies, the findings suggest that the sequence of combining these agents may have different effects on kidney preservation.

Our results are consistent with other observational studies. In one study, a post hoc subgroup analysis of individuals with type 2 diabetes who also had CKD (*N* = 438) with combined SGLT2i and GLP‐1 RA therapy, after weighting the outcomes (≥ 50% reduction in eGFR and/or progression to macroalbuminuria) in a win‐ratio analysis, people treated with a GLP‐1 RA preceding an SGLT2i had more positive kidney outcomes than those treated with an SGLT2i prior to GLP‐1 RA (odds ratio 1.83, 95% CI 1.71 to 1.95) [34]. However, it was unclear whether the benefits could be credited to the addition of an SGLT2i, or whether GLP‐1 RAs enhance the treatment effect of the SGLT2i.

In another observational study, combination therapy was associated with a lower risk of MACE compared with either SGLT2i or GLP‐1 RA monotherapy, and a lower risk of serious clinical kidney events compared with GLP‐1 RA monotherapy (hazard ratio [HR] 0.43, 95% CI 0.23 to 0.80) [17]. But there was no difference in the risk of serious kidney events compared with SGLT2i monotherapy (HR 0.67, 95% CI 0.32 to 1.41).

A meta‐analysis of cohort studies showed that combination therapy was associated with a lower risk of MACE (risk ratio [RR] 0.56, 95% CI 0.43 to 0.71) and adverse kidney outcomes (RR 0.48, 95% CI 0.32 to 0.73) compared with either SGLT2i or GLP‐1 RA alone (monotherapy arms were pooled into a single comparator group) [35]. Separate analysis according to the type of drug class comparator (SGLT2i or GLP‐1 RA monotherapy) showed consistent findings for the effect of combination therapy on MACE. However, the equivalent subgroup analysis was not reported for the composite kidney endpoint.

To date, only one reported prospective randomised trial has evaluated the effect of add‐on treatment with SGLT2i or GLP‐1 RA in patients with type 2 diabetes and CKD. The Evaluate Renal Function with Semaglutide Once Weekly (FLOW) trial assessed the effect of semaglutide on kidney outcomes in people with type 2 diabetes and CKD [36]. A subgroup analysis found no clear difference in effect on eGFR slope according to background treatment with an SGLT2i, though the analysis was underpowered.

### 4.3 UACR In Early Diabetic Kidney Disease

We found a reduction in UACR at 2‐years with combination use of SGLT2i plus GLP‐1 RA compared with GLP‐1 RA monotherapy. Albuminuria is a key marker for early onset of diabetic kidney disease, as well as a risk factor for cardiovascular disease [37, 38, 39]. In type 2 diabetes, the eGFR typically declines at −1.5 to −4.0 mL/min/1.73 m^2^ per year. Hence, a 2 to 3 year study may not be long enough to detect changes in eGFR, and longer follow‐up is required. Conversely, in type 2 diabetes, elevated UACR often precedes a decline in eGFR, indicating kidney damage [40].

### 4.4 Baseline Characteristics of the Study Population

Most of our study population had an eGFR ≥ 60 mL/min/1.73 m^2^ at baseline. By contrast, two previous observational cohort studies that assessed kidney outcomes in patients treated with SGLT2i plus GLP‐1 RA in combination versus monotherapy had a minimum prevalence for baseline CKD of 9% and 10% [16, 17]. In another retrospective cohort study—of individuals that were overweight or obese and with type 2 diabetes and heart failure with preserved ejection fraction—people treated with GLP‐1 RA added to SGLT2i versus SGLT2i alone had reduced risk of acute kidney injury and renal replacement therapy [15]. Our study provides further insights on the kidney effects of SGLT2i plus GLP‐1 RA combination therapy in a broader and more diverse population of people with type 2 diabetes.

### 4.5 Strengths and Limitations

The substantial size of the RSC database provided large samples in each matched cohort for sufficient statistical power [20]. This allowed us to perform multiple subgroup analyses and sensitivity analyses to corroborate our findings, which were largely consistent with the primary analysis.

Data completeness of the RSC database is high, likely due to the Quality and Outcomes Framework (QOF) [21]. QOF is a pay‐for‐performance incentive scheme introduced to UK primary care in 2004 to improve clinical coding [41]. Data quality within the RSC network is further enriched by a dedicated team of practice liaison officers, who work closely with the practices, providing training and support, including data coding [18].

The study was performed as an intention‐to‐treat analysis to mimic the design of a randomised controlled trial. However, this assumes that participants adhere to taking their medications, which is challenging to control for in observational studies. We could not adjust for adherence as a sensitivity ‘on‐treatment’ analysis as the RSC data do not contain specific information on ‘directions of use’ to allow calculation of drug dose.

The RSC database does not include information on dispensed data to indicate that medications are being collected and taken (as prescribed). Other residual confounding factors may have contributed, including lifestyle and dietary changes, which are not recorded in patients' CMRs. However, any substantial confounding was largely reduced from the propensity score matching and additional adjustments on potential confounders that remained unbalanced between combination and either monotherapy.

Missing data may have impacted upon the findings. Our sensitivity analysis to assign missing data to ‘unknown categories’ in covariates during matching showed results consistent with the main findings. Yet, we were unable to deal with missing data in the outcome variables. The COVID‐19 pandemic may have also impacted on missing data, as face‐to‐face consultations reduced substantially because of social restrictions in the UK [27]. However, our sensitivity analysis to include only individuals initiated an SGLT2i and/or GLP‐1 RA before the COVID‐19 pandemic was consistent with the findings from our main analysis.

The data were extracted at a time when most people with type 2 diabetes were initiated on medications to improve their glycaemic control, rather than protect their kidney function [2]. As a result, almost the entire cohort had an eGFR ≥ 60 mL/min/1.73 m^2^ at baseline. This precluded subgroup analysis according to CKD status. The duration of follow‐up may not have been long enough to detect changes in eGFR in those with a well‐preserved eGFR at baseline. Moreover, the database is not linked to hospital data, which may have potentially included more patients with diabetes and established CKD. An updated analysis with a newer dataset may provide more insights as guidelines now recommend the medications we studied in people with CKD [42, 43, 44].

## 5 Conclusions

This is one of the first large real‐world studies assessing biochemical measures of kidney function following prescription of the combination of SGLT2i plus GLP‐1 RA therapy in the management of hyperglycaemia in a type 2 diabetes cohort with predominantly normal baseline eGFR. The addition of an SGLT2i to baseline GLP‐1 RA therapy may be more effective in preserving kidney function in the first 2 years than GLP‐1 RA monotherapy. When the other combination was assessed (combining GLP‐1 RA with baseline SGLT2i therapy), there was no additional kidney benefit. Future studies (including observational) should be at least 2 years to investigate potential combined medication effects on kidney outcomes, including the impact of drug initiation sequence.

## Author Contributions

M.D.F. conceptualised the study. W.H., M.D.F. and A.K.F. designed the study with input from all authors. A.K.F. curated the required variables from the clinical codes, and X.F. performed the data extraction. W.H. performed the data analyses with statistical input from M.J. and J.M.O.‐M. Drafting of the manuscript was led by W.H., with review and contributions from all authors. All authors approved the final manuscript. W.H. is the guarantor of this work and accepts responsibility for conduct of the study, access to the data, and the decision to submit and publish the manuscript. W.H. attests that all listed authors meet authorship criteria and that no others meeting the criteria have been omitted.

## Funding

This study was funded by an MSD (Merck Sharp & Dohme Ltd) Investigator Studies Programme grant (MISP 60830). The study funder was not involved in the design of the study; the collection, analysis, and interpretation of data; writing the report; and did not impose any restrictions regarding the publication of the report.

## Conflicts of Interest

W.H. has had part of his academic salary funded from grant awards with Eli Lilly and Co., Novo Nordisk Ltd., AstraZeneca UK Ltd., and Merck Sharp & Dohme Ltd. A.K.F. has received travel expenses from AstraZeneca. S.d.L. has received grants through the Nuffield Department of Primary Care Health Sciences, University of Oxford for investigator‐led studies in diabetes and cardio‐metabolic disease from GlaxoSmithKline, Eli Lilly and Co., Novo Nordisk Ltd., Sanofi, and Merck Sharp & Dohme Ltd. M.B.W. has received grant funding from Sanofi, Eli Lilly and Co., and speaker fees from AstraZeneca and Merck Sharp & Dohme Ltd. D.C.W. has received honoraria and/or consultancy fees in the last 2 years from AstraZeneca, Astellas, Bayer, Boehringer Ingelheim, CSL, Dimetrix, GSK, Menarini, MSD, Pathaly, ProKidney, PureSpring, Vertex, and Vifor. M.D.F. was awarded a grant from Merck Sharp & Dohme Ltd. through the Nuffield Department of Primary Care Health Sciences, University of Oxford for this study. M.D.F. has received personal speaker fees from Sanofi and Menarini. All other authors declare no competing interests.

## Supporting information


**Supporting Information: 1** dom70946‐sup‐0001‐Supplementary_1.docx

Supporting Information: 2
**Figure S1:** Time‐based exposure sets for the base cohort of SGLT2i users.
**Figure S2:** Flowchart for the development of the SGLT2i plus GLP‐1 RA combination compared with the SGLT2i monotherapy new user cohorts.
**Table S1:** Baseline characteristics of the SGLT2i plus GLP‐1 RA combination users and SGLT2i users before propensity score matching.
**Figure S3:** Measure of balance across covariates between SGLT2i plus GLP‐1 RA combination and SGLT2i monotherapy before (blue circles) and after (red circles) matching.
**Figure S4:** Frequency of SGLT2i plus GLP‐1 RA combination users and matched SGLT2i monotherapy users, by duration of follow up (years).
**Table S2:** Effect of SGLT2i plus GLP‐1 RA combination therapy on mean absolute change in eGFR (from baseline to 1‐ and 2‐years follow‐up) compared with SGLT2i monotherapy, stratified by UACR subgroup.
**Table S3:** Effect of SGLT2i plus GLP‐1 RA combination therapy on mean absolute change in eGFR (from baseline to 1‐ and 2‐years follow‐up) compared with SGLT2i monotherapy, stratified by HbA1c subgroup.
**Table S4:** Effect of SGLT2i plus GLP‐1 RA combination therapy on mean absolute change in eGFR (from baseline to 1‐ and 2‐years follow‐up) compared with SGLT2i monotherapy, stratified by previous use of RAS inhibitors.
**Table S5:** Effect of SGLT2i plus GLP‐1 RA combination therapy on mean absolute change in eGFR (from baseline to 1‐ and 2‐years follow‐up) compared with SGLT2i monotherapy, including only individuals that were initiated therapies up to the 31 December 2019.
**Table S6:** Effect of SGLT2i plus GLP‐1 RA combination therapy on mean absolute change in eGFR (from baseline to 1‐ and 2‐years follow‐up) compared with SGLT2i monotherapy, including all individuals in the propensity score matching process (regardless of missing data).
**Figure S5:** Flowchart for the development of the SGLT2i plus GLP‐1 RA combination compared with the GLP‐1 RA monotherapy new user cohorts.
**Table S7:** Baseline characteristics of the SGLT2i plus GLP‐1 RA combination users and GLP‐1 RA users before propensity score matching.
**Figure S6:** Measure of balance across covariates between SGLT2i plus GLP‐1 RA combination and GLP‐1 RA monotherapy before (blue circles) and after (red circles) matching.
**Figure S7:** Frequency of SGLT2i plus GLP‐1 RA combination users and matched GLP‐1 RA monotherapy users, by duration of follow up (years).
**Table S8:** Effect of SGLT2i plus GLP‐1 RA combination therapy on mean absolute change in eGFR (from baseline to 1‐ and 2‐years follow‐up) compared with GLP‐1 RA monotherapy, stratified by UACR subgroup.
**Table S9:** Effect of SGLT2i plus GLP‐1 RA combination therapy on mean absolute change in eGFR (from baseline to 1‐ and 2‐years follow‐up) compared with GLP‐1 RA monotherapy, stratified by HbA1c subgroup.
**Table S10:** Effect of SGLT2i plus GLP‐1 RA combination therapy on mean absolute change in eGFR (from baseline to 1‐ and 2‐years follow‐up) compared with GLP‐1 RA monotherapy, stratified by previous use of RAS inhibitors.
**Table S11:** Effect of SGLT2i plus GLP‐1 RA combination therapy on mean absolute change in eGFR (from baseline to 1‐ and 2‐years follow‐up) compared with GLP‐1 RA monotherapy, including only individuals that were initiated therapies up to the 31 December 2019.
**Table S12:** Effect of SGLT2i plus GLP‐1 RA combination therapy on mean absolute change in eGFR (from baseline to 1‐ and 2‐years follow‐up) compared with GLP‐1 RA monotherapy, including all individuals in matching process, regardless of missing data.

## Data Availability

Access to the pseudonymised patient‐level data is considered on a project‐by‐project basis. Researchers wishing to access the data are required to submit a data request form, study protocol and confirmation of ethical approval to the scientific committee at the University of Oxford. Following approval, researchers need to complete mandatory information governance training prior to being given access to the requested data. Researchers are required to work on the data from the secure servers at the University of Oxford. Patient‐level data cannot be taken out of the secure network.
