# Supplementary material for: Combining Sodium–Glucose Co‐Transporter‐2 Inhibitor Plus Glucagon‐Like Peptide‐1 Receptor Agonist for Glucose‐Lowering in Type 2 Diabetes: Effects of Drug Initiation Sequence on Kidney Function in Real‐World Clinical Practice (CombiKid Study)
Source: Diabetes Obes Metab. 2026 Jun 16;28(9):7950–63. doi: 10.1111/dom.70946 (PMC13448856; doi:10.1111/dom.70946)
Supplement: Supplementary file 1 [file DOM-28-7950-s001.docx]

# **Supplementary 2**

## Tables and figures

**Figure S1. Time-based exposure sets for the base cohort of SGLT2i users.** “Time zero” represents the first prescription date for an SGLT2i. Each orange circle represents a new SGLT2i prescription, a blue square indicates when a GLP-1 RA is added (combination user), and the grey hashed borders indicate that a monotherapy user’s new SGLT2i prescription falls with a 90-day interval of a combination user.

**Figure S2. Flowchart for the development of the SGLT2i plus GLP-1 RA combination compared to the SGLT2i monotherapy new user cohorts.**

**Table S1. Baseline characteristics of the SGLT2i plus GLP-1 RA combination users and SGLT2i users before propensity score matching.**

| **Characteristics** | **SGLT2i plus GLP-1 RA combination**  **(N = 11,350)** | **SGLT2i monotherapy**  **(N = 49,297)** | **Std. diff.** |
| --- | --- | --- | --- |
| Median (IQR) age, years | 61.6 (55.5 - 68.2) | 63.0 (56.1 - 70.1) | 0.139 |
| Male | 6,287 (55.4) | 31,614 (64.1) | 0.179 |
| ***Ethnicity*** |  |  |  |
| White | 9,728 (85.7) | 38,277 (77.6) | 0.210 |
| Asian | 606 (5.3) | 4,944 (10.0) | 0.178 |
| Black | 258 (2.3) | 1,683 (3.4) | 0.066 |
| Mixed | 75 (0.7) | 450 (0.9) | 0.022 |
| Other | 94 (0.8) | 441 (0.9) | 0.011 |
| Unknown | 589 (5.2) | 3,502 (7.1) | 0.079 |
| ***IMD Quintile*** |  |  |  |
| 5 (least deprived) | 1,993 (17.6) | 8,996 (18.2) | 0.016 |
| 4 | 2,266 (20.0) | 9,872 (20.0) | 0.000 |
| 3 | 2,220 (19.6) | 9,498 (19.3) | 0.008 |
| 2 | 2,290 (20.2) | 10,247 (20.8) | 0.015 |
| 1 (most deprived) | 2,581 (22.7) | 10,684 (21.7) | 0.032 |
| Median (IQR) duration of diabetes, years | 9.6 (5.9 - 13.8) | 7.7 (3.6 - 12.3) | 0.024 |
| ***Duration of diabetes categories*** |  |  |  |
| <1 year | 154 (1.4) | 5,154 (10.5) | 0.392 |
| 1-5 years | 5,329 (47.0) | 18,054 (36.6) | 0.212 |
| 5-10 years | 2,033 (17.9) | 11,385 (23.1) | 0.129 |
| ≥10 years | 3,834 (33.8) | 14,704 (29.8) | 0.086 |
| Median (IQR) BMI, kg/m^2^ | 34.1 (30.7 - 38.5) | 31.1 (27.8 - 35.4) | 0.481 |
| ***BMI categories*** |  |  |  |
| Underweight | <5 (0.0) | 81 (0.2) | 0.063 |
| Normal weight | 247 (2.2) | 4,222 (8.6) | 0.286 |
| Overweight | 1,936 (17.1) | 14,868 (30.2) | 0.312 |
| Obese | 8,866 (78.1) | 27,386 (55.6) | 0.492 |
| Unknown | 300 (2.6) | 2,740 (5.6) | 0.152 |
| Median (IQR) HbA1c, mmol/mol | 74.0 (65.0 - 86.0) | 71.0 (62.0 - 84.0) | 0.177 |
| Median (IQR) HbA1c, % | 8.9 (8.1 - 10.0) | 8.6 (7.8 - 9.8) | 0.153 |
| ***HbA1c categories*** |  |  |  |
| <53 mmol/mol (<7%) | 369 (3.3) | 4,426 (9.0) | 0.268 |
| 53-63 mmol/mol (7-8%) | 1,877 (16.5) | 10,216 (20.7) | 0.108 |
| 64-74 mmol/mol (8-9%) | 3,536 (31.2) | 13,684 (27.8) | 0.075 |
| 75-85 mmol/mol (9-10%) | 2,681 (23.6) | 8,912 (18.1) | 0.136 |
| >=86 mmol/mol (10%) | 2,853 (25.1) | 11,114 (22.5) | 0.061 |
| Unknown | 34 (0.3) | 945 (1.9) | 0.154 |
| Median (IQR) UACR, mg/mmol | 1.8 (0.9 - 3.5) | 1.5 (0.7 - 3.3) | 0.008 |
| ***UACR categories*** |  |  |  |
| <3 mg/mmol | 5,687 (50.1) | 24,601 (49.9) | 0.004 |
| 3-30 mg/mmol | 318 (2.8) | 1,331 (2.7) | 0.006 |
| >30 mg/mmol | 2,360 (20.8) | 8,854 (18.0) | 0.071 |
| Unknown | 2,985 (26.3) | 14,511 (29.4) | 0.069 |
| Median (IQR) eGFR, mL/min/1.73 m^2^ | 90.5 (79.3 - 97.8) | 89.5 (78.2 - 96.6) | 0.082 |
| ***eGFR categories*** |  |  |  |
| <60 | 686 (6.0) | 3,258 (6.6) | 0.025 |
| >=60 | 10,620 (93.6) | 45,624 (92.5) | 0.043 |
| Unknown | 44 (0.4) | 415 (0.8) | 0.052 |
| ***Comorbidities*** |  |  |  |
| Hypertension | 7,797 (68.7) | 31,696 (64.3) | 0.093 |
| CVD | 2,249 (19.8) | 10,860 (22.0) | 0.054 |
| Heart failure | 577 (5.1) | 5,121 (10.4) | 0.200 |
| Haemorrhagic stroke | 66 (0.6) | 325 (0.7) | 0.010 |
| Neuropathy | 610 (5.4) | 1,989 (4.0) | 0.063 |
| Retinopathy | 3,963 (34.9) | 14,809 (30.0) | 0.104 |
| Valvular heart disease | 303 (2.7) | 1,995 (4.0) | 0.077 |
| ***Medications*** |  |  |  |
| ACE inhibitor | 2,784 (24.5) | 11,993 (24.3) | 0.005 |
| ARB | 7,796 (68.7) | 31,887 (64.7) | 0.085 |
| NSAID | 9,545 (84.1) | 38,702 (78.5) | 0.144 |
| Acarbose | 87 (0.8) | 301 (0.6) | 0.019 |
| Beta blocker | 4,615 (40.7) | 20,177 (40.9) | 0.005 |
| Diuretic | 4,696 (41.4) | 19,751 (40.1) | 0.027 |
| Metformin | 11,241 (99.0) | 45,567 (92.4) | 0.331 |
| Statin | 10,362 (91.3) | 42,511 (86.2) | 0.161 |
| Sulphonylurea | 7,055 (62.2) | 23,667 (48.0) | 0.287 |
| DPP-4 inhibitor | 7,481 (65.9) | 21,917 (44.5) | 0.442 |
| Insulin | 1,878 (16.5) | 4,933 (10.0) | 0.194 |

ACE = angiotensin-converting enzyme; ARB = angiotensin receptor blockers; BMI = body mass index; CVD = cardiovascular disease; DPP-4 = dipeptidyl peptidase 4; eGFR = estimated glomerular filtration rate; HbA1c = glycated haemoglobin; IMD = Index of Multiple Deprivation; IQR = interquartile range; NSAID = non-steroidal anti-inflammatory drug; Std. diff. = standardised difference; UACR = urine albumin to creatinine ratio

**Figure S3. Measure of balance across covariates between SGLT2i plus GLP-1 RA combination and SGLT2i monotherapy before (blue circles) and after (red circles) matching. Standardised differences <0.1 indicate good balance.**

**Figure S4.** **Frequency of SGLT2i plus GLP-1 RA combination users and matched SGLT2i monotherapy users, by duration of follow up (years).**

**Table S2.** **Effect of SGLT2i plus GLP-1 RA combination therapy on mean absolute change in eGFR (from baseline to 1- and 2-years follow-up) compared to SGLT2i monotherapy, stratified by UACR subgroup**

| **Outcome**  Exposure group | **No. with a measurement** | **Mean outcome** | **Crude Mean difference (95% CI)** | **Adjusted Mean difference (95% CI)†** |
| --- | --- | --- | --- | --- |
| **eGFR - 1 year (mL/min/1.73 m^2^)** | | | | |
| ***Subgroup: UACR <3 mg/mmol*** | | | | |
| SGLT2i + GLP-1 RA | 3,646 | -3.4 | 0.6 (-0.09 to 1.29) | 0.4 (-0.15 to 0.98) |
| SGLT2i | 3,582 | -4.0 | 0 (Reference) | 0 (Reference) |
| ***Subgroup: UACR ≥3 mg/mmol*** | | | | |
| SGLT2i + GLP-1 RA | 1,205 | -7.2 | 0.2 (-1.25 to 1.59) | -0.1 (-1.26 to 1.13) |
| SGLT2i | 977 | -7.4 | 0 (Reference) | 0 (Reference) |
| **eGFR - 2 years (mL/min/1.73 m^2^)** | | | | |
| ***Subgroup: UACR <3 mg/mmol*** | | | | |
| SGLT2i + GLP-1 RA | 2,481 | -4.1 | -0.1 (-0.90 to 0.79) | -0.4 (-1.05 to 0.34) |
| SGLT2i | 2,562 | -4.0 | 0 (Reference) | 0 (Reference) |
| ***Subgroup: UACR ≥3 mg/mmol*** | | | | |
| SGLT2i + GLP-1 RA | 833 | -8.2 | 0.2 (-1.53 to 2.02) | -0.5 (-2.04 to 1.02) |
| SGLT2i | 677 | -8.5 | 0 (Reference) | 0 (Reference) |

**†**Further adjusted for age, sex, ethnicity, socioeconomic status, body mass index (BMI), hypertension, cardiovascular disease, heart failure, haemorrhagic stroke, neuropathy, retinopathy, valvular heart disease, angiotensin-converting enzyme (ACE) inhibitors, angiotensin receptor blockers (ARB), diuretics, beta blockers, statins, acarbose, dipeptidyl peptidase-4 (DPP-4) inhibitors, insulin, metformin, sulphonylureas, thiazolidinediones, non-steroidal anti-inflammatory drugs (NSAID), duration of diabetes, HbA1c, eGFR, and UACR.

CI = confidence interval; eGFR = estimated glomerular filtration rate, HbA1c = glycated haemoglobin; UACR = urine albumin creatinine ratio

**Table S3.** **Effect of SGLT2i plus GLP-1 RA combination therapy on mean absolute change in eGFR (from baseline to 1- and 2-years follow-up) compared to SGLT2i monotherapy, stratified by HbA1c subgroup**

| **Outcome**  Exposure group | **No. with a measurement** | **Mean outcome** | **Crude Mean difference (95% CI)** | **Adjusted Mean difference (95% CI)†** |
| --- | --- | --- | --- | --- |
| **eGFR - 1 year (mL/min/1.73 m^2^)** | | | | |
| ***Subgroup: HbA1c <53 mmol/mol (<7%)*** | | | | |
| SGLT2i + GLP-1 RA | 381 | -4.0 | 1.8 (-0.12 to 3.76) | 1.3 (-0.28 to 2.94) |
| SGLT2i | 660 | -5.8 | 0 (Reference) | 0 (Reference) |
| ***Subgroup: HbA1c ≥53 and <64 mmol/mol (≥7 and <8%)*** | | | | |
| SGLT2i + GLP-1 RA | 884 | -4.1 | 0.6 (-0.70 to 1.85) | 0.6 (-0.42 to 1.64) |
| SGLT2i | 1388 | -4.7 | 0 (Reference) | 0 (Reference) |
| ***Subgroup: HbA1c ≥64 mmol/mol*** ***(≥8%)*** | | | | |
| SGLT2i + GLP-1 RA | 3586 | -4.4 | 0.0 (-0.78 to 0.82) | 0.1 (-0.57 to 0.71) |
| SGLT2i | 2511 | -4.5 | 0 (Reference) | 0 (Reference) |
| **eGFR - 2 years**  **(mL/min/1.73 m^2^)** | | | | |
| ***Subgroup: HbA1c <53 mmol/mol (<7%)*** | | | | |
| SGLT2i + GLP-1 RA | 209 | -5.0 | 0.5 (-2.21 to 3.29) | -1.1 (-3.3 to 1.15) |
| SGLT2i | 422 | -5.6 | 0 (Reference) | 0 (Reference) |
| ***Subgroup: HbA1c ≥53 and <64 mmol/mol (≥7 and <8%)*** | | | | |
| SGLT2i + GLP-1 RA | 606 | -4.4 | 0.7 (-0.85 to 2.31) | 0.7 (-0.57 to 2.04) |
| SGLT2i | 970 | -5.2 | 0 (Reference) | 0 (Reference) |
| ***Subgroup: HbA1c ≥64 mmol/mol*** | | | | |
| SGLT2i + GLP-1 RA | 2499 | -5.3 | -0.6 (-1.56 to 0.36) | -0.6 (-1.42 to 0.13) |
| SGLT2i | 1847 | -4.7 | 0 (Reference) | 0 (Reference) |

**†**Further adjusted for age, sex, ethnicity, socioeconomic status, body mass index (BMI), hypertension, cardiovascular disease, heart failure, haemorrhagic stroke, neuropathy, retinopathy, valvular heart disease, angiotensin-converting enzyme (ACE) inhibitors, angiotensin receptor blockers (ARB), diuretics, beta blockers, statins, acarbose, dipeptidyl peptidase-4 (DPP-4) inhibitors, insulin, metformin, sulphonylureas, thiazolidinediones, non-steroidal anti-inflammatory drugs (NSAID), duration of diabetes, HbA1c, eGFR, and UACR.

CI = confidence interval; eGFR = estimated glomerular filtration rate, HbA1c = glycated haemoglobin; UACR = urine albumin creatinine ratio

**Table S4.** **Effect of SGLT2i plus GLP-1 RA combination therapy on mean absolute change in eGFR (from baseline to 1- and 2-years follow-up) compared to SGLT2i monotherapy, stratified by previous use of RAS inhibitors**

| **Outcome**  Exposure group | **No. with a measurement** | **Mean outcome** | **Crude Mean difference (95% CI)** | **Adjusted Mean difference (95% CI)†** |
| --- | --- | --- | --- | --- |
| **eGFR - 1 year (mL/min/1.73 m^2^)** | | | | |
| ***Subgroup: RAS inhibitor*** | | | | |
| SGLT2i + GLP-1 RA | 3,325 | -5.5 | 0.6 (-0.23 to 1.33) | 0.4 (-0.25 to 1.04) |
| SGLT2i | 3,145 | -6.0 | 0 (Reference) | 0 (Reference) |
| ***Subgroup: No RAS inhibitor*** | | | | |
| SGLT2i + GLP-1 RA | 1,526 | -1.9 | 0.0 (-1.08 to 0.99) | 0.3 (-0.53 to 1.13) |
| SGLT2i | 1,414 | -1.8 | 0 (Reference) | 0 (Reference) |
| **eGFR - 2 years**  **(mL/min/1.73 m^2^)** | | | | |
| ***Subgroup: RAS inhibitor*** | | | | |
| SGLT2i + GLP-1 RA | 2,261 | -6.6 | -0.4 (-1.37 to 0.52) | -0.5 (-1.25 to 0.35) |
| SGLT2i | 2,235 | -6.2 | 0 (Reference) | 0 (Reference) |
| ***Subgroup: No RAS inhibitor*** | | | | |
| SGLT2i + GLP-1 RA | 1,053 | -2.0 | 0.3 (-1.01 to 1.59) | 0.0 (-1.04 to 1.01) |
| SGLT2i | 1,004 | -2.3 | 0 (Reference) | 0 (Reference) |

**†**Further adjusted for age, sex, ethnicity, socioeconomic status, body mass index (BMI), hypertension, cardiovascular disease, heart failure, haemorrhagic stroke, neuropathy, retinopathy, valvular heart disease, diuretics, beta blockers, statins, acarbose, dipeptidyl peptidase-4 (DPP-4) inhibitors, insulin, metformin, sulphonylureas, thiazolidinediones, non-steroidal anti-inflammatory drugs (NSAID), duration of diabetes, HbA1c, eGFR, and UACR.

CI = confidence interval; eGFR = estimated glomerular filtration rate, HbA1c = glycated haemoglobin; RAS = Renin–angiotensin system; UACR = urine albumin creatinine ratio

**Table S5.** **Effect of SGLT2i plus GLP-1 RA combination therapy on mean absolute change in eGFR (from baseline to 1- and 2-years follow-up) compared to SGLT2i monotherapy, including only individuals that were initiated therapies up to the 31^st^ December 2019**

| **Outcome**  Exposure group | **No. with a measurement** | **Mean outcome** | **Crude Mean difference (95% CI)** | **Adjusted Mean difference (95% CI)†** |
| --- | --- | --- | --- | --- |
| **eGFR - 1 year**  **(mL/min/1.73 m^2^)** | | | | |
| SGLT2i + GLP-1 RA | 2,534 | -3.2 | -0.2 (-1.01 to 0.65) | -0.3 (-0.93 to 0.41) |
| SGLT2i | 2,419 | -3.0 | 0 (Reference) | 0 (Reference) |
| **eGFR - 2 years**  **(mL/min/1.73 m^2^)** | | | | |
| SGLT2i + GLP-1 RA | 2,344 | -4.6 | -0.5 (-1.38 to 0.43) | -0.9 (-1.68 to -0.20) |
| SGLT2i | 2,264 | -4.1 | 0 (Reference) | 0 (Reference) |

**†**Further adjusted for age, sex, ethnicity, socioeconomic status, body mass index (BMI), hypertension, cardiovascular disease, heart failure, haemorrhagic stroke, neuropathy, retinopathy, valvular heart disease, angiotensin-converting enzyme (ACE) inhibitors, angiotensin receptor blockers (ARB), diuretics, beta blockers, statins, acarbose, dipeptidyl peptidase-4 (DPP-4) inhibitors, insulin, metformin, sulphonylureas, thiazolidinediones, non-steroidal anti-inflammatory drugs (NSAID), duration of diabetes, HbA1c, eGFR, and UACR.

CI = confidence interval; eGFR = estimated glomerular filtration rate, HbA1c = glycated haemoglobin; UACR = urine albumin creatinine ratio

**Table S6.** **Effect of SGLT2i plus GLP-1 RA combination therapy on mean absolute change in eGFR (from baseline to 1- and 2-years follow-up) compared to SGLT2i monotherapy, including all individuals in the propensity score matching process (regardless of missing data)**

| **Outcome**  Exposure group | **No. with a measurement** | **Mean outcome** | **Crude Mean difference (95% CI)** | **Adjusted Mean difference (95% CI)†** |
| --- | --- | --- | --- | --- |
| **eGFR - 1 year**  **(mL/min/1.73 m^2^)** | | | | |
| SGLT2i + GLP-1 RA | 5,324 | -4.2 | 0.1 (-0.53 to 0.70) | 0.3 (-0.13 to 0.83) |
| SGLT2i | 5,021 | -4.2 | 0 (Reference) | 0 (Reference) |
| **eGFR - 2 years**  **(mL/min/1.73 m^2^)** | | | | |
| SGLT2i + GLP-1 RA | 3,656 | -4.9 | -0.4 (-1.16 to 0.33) | -0.1 (-0.75 to 0.45) |
| SGLT2i | 3,542 | -4.5 | 0 (Reference) | 0 (Reference) |

**†**Further adjusted for age, sex, ethnicity, socioeconomic status, body mass index (BMI), hypertension, cardiovascular disease, heart failure, haemorrhagic stroke, neuropathy, retinopathy, valvular heart disease, angiotensin-converting enzyme (ACE) inhibitors, angiotensin receptor blockers (ARB), diuretics, beta blockers, statins, acarbose, dipeptidyl peptidase-4 (DPP-4) inhibitors, insulin, metformin, sulphonylureas, thiazolidinediones, non-steroidal anti-inflammatory drugs (NSAID), duration of diabetes, HbA1c, eGFR, and UACR.

CI = confidence interval; eGFR = estimated glomerular filtration rate, HbA1c = glycated haemoglobin; UACR = urine albumin creatinine ratio

**Figure S5. Flowchart for the development of the SGLT2i plus GLP-1 RA combination compared to the GLP-1 RA monotherapy new user cohorts**

**Table S7.** **Baseline characteristics of the SGLT2i plus GLP-1 RA combination users and GLP-1 RA users before propensity score matching.**

| **Characteristics** | **SGLT2i plus GLP-1 RA combination**  **(N = 8,603)** | **GLP-1 RA monotherapy**  **(N = 11,581)** | **Std. diff.** |
| --- | --- | --- | --- |
| Median (IQR) age, years | 61.3 (55.2 - 67.7) | 63.5 (56.0 - 70.8) | 0.196 |
| Male | 4,747 (55.2) | 5,887 (50.8) | 0.087 |
| ***Ethnicity*** |  |  |  |
| White | 7,390 (85.9) | 9,772 (84.4) | 0.042 |
| Asian | 443 (5.1) | 488 (4.2) | 0.043 |
| Black | 173 (2.0) | 312 (2.7) | 0.046 |
| Mixed | 56 (0.7) | 83 (0.7) | 0.000 |
| Other | 74 (0.9) | 50 (0.4) | 0.062 |
| Unknown | 467 (5.4) | 876 (7.6) | 0.089 |
| ***IMD Quintile*** |  |  |  |
| 5 (least deprived) | 1,480 (17.2) | 1,881 (16.2) | 0.027 |
| 4 | 1,721 (20.0) | 2,141 (18.5) | 0.038 |
| 3 | 1,684 (19.6) | 2,340 (20.2) | 0.015 |
| 2 | 1,765 (20.5) | 2,411 (20.8) | 0.007 |
| 1 (most deprived) | 1,953 (22.7) | 2,808 (24.2) | 0.035 |
| Median (IQR) duration of diabetes, years | 9.3 (5.6 - 13.6) | 8.5 (3.9 - 13.6) | 0.102 |
| ***Duration of diabetes categories*** |  |  |  |
| <1 year | 140 (1.6) | 1,196 (10.3) | 0.374 |
| 1-5 years | 3,899 (45.3) | 4,889 (42.2) | 0.063 |
| 5-10 years | 1,638 (19.0) | 2,391 (20.6) | 0.040 |
| ≥10 years | 2,926 (34.0) | 3,105 (26.8) | 0.157 |
| Median (IQR) BMI, kg/m^2^ | 34.4 (30.9 - 38.9) | 36.3 (32.4 - 41.4) | 0.293 |
| ***BMI categories*** |  |  |  |
| Underweight | <5 (0.0) | 6 (0.1) | 0.045 |
| Normal weight | 175 (2.0) | 146 (1.3) | 0.055 |
| Overweight | 1,385 (16.1) | 1,147 (9.9) | 0.185 |
| Obese | 6,818 (79.3) | 9,722 (83.9) | 0.119 |
| Unknown | 224 (2.6) | 560 (4.8) | 0.117 |
| Median (IQR) HbA1c, mmol/mol | 74.0 (66.0 - 86.0) | 74.0 (63.0 - 88.0) | 0.055 |
| Median (IQR) HbA1c, % | 8.9 (8.2 - 10.0) | 8.9 (7.9 - 10.2) | 0.000 |
| ***HbA1c categories*** |  |  |  |
| <53 mmol/mol (<7%) | 271 (3.2) | 1,141 (9.9) | 0.273 |
| 53-63 mmol/mol (7-8%) | 1,372 (15.9) | 1,774 (15.3) | 0.017 |
| 64-74 mmol/mol (8-9%) | 2,661 (30.9) | 2,802 (24.2) | 0.150 |
| 75-85 mmol/mol (9-10%) | 2,041 (23.7) | 2,371 (20.5) | 0.077 |
| >=86 mmol/mol (10%) | 2,231 (25.9) | 3,189 (27.5) | 0.036 |
| Unknown | 27 (0.3) | 304 (2.6) | 0.193 |
| Median (IQR) UACR, mg/mmol | 1.7 (0.8 - 3.5) | 1.9 (0.8 - 4.5) | 0.103 |
| ***UACR categories*** |  |  |  |
| <3 mg/mmol | 4,366 (50.7) | 5,530 (47.8) | 0.058 |
| 3-30 mg/mmol | 1,765 (20.5) | 2,362 (20.4) | 0.002 |
| >30 mg/mmol | 242 (2.8) | 536 (4.6) | 0.095 |
| Unknown | 2,230 (25.9) | 3,153 (27.2) | 0.029 |
| Median (IQR) eGFR, mL/min/1.73 m^2^ | 90.8 (79.4 - 97.9) | 84.9 (64.7 - 96.0) | 0.443 |
| ***eGFR categories*** |  |  |  |
| <60 mL/min/1.73 m^2^ | 485 (5.6) | 2,294 (19.8) | 0.436 |
| ≥60 mL/min/1.73 m^2^ | 8,082 (93.9) | 9,065 (78.3) | 0.463 |
| Unknown | 36 (0.4) | 222 (1.9) | 0.141 |
| ***Comorbidities*** |  |  |  |
| Hypertension | 5,870 (68.2) | 8,252 (71.3) | 0.066 |
| CVD | 1,726 (20.1) | 2,736 (23.6) | 0.086 |
| Heart failure | 386 (4.5) | 800 (6.9) | 0.105 |
| Haemorrhagic stroke | 53 (0.6) | 97 (0.8) | 0.026 |
| Neuropathy | 464 (5.4) | 679 (5.9) | 0.020 |
| Retinopathy | 3,025 (35.2) | 3,975 (34.3) | 0.018 |
| Valvular heart disease | 230 (2.7) | 405 (3.5) | 0.048 |
| ***Medications*** |  |  |  |
| ACE inhibitor | 2,066 (24.0) | 3,105 (26.8) | 0.064 |
| ARB | 5,911 (68.7) | 8,082 (69.8) | 0.023 |
| NSAID | 7,231 (84.1) | 9,481 (81.9) | 0.058 |
| Acarbose | 75 (0.9) | 152 (1.3) | 0.042 |
| Beta blocker | 3,482 (40.5) | 5,229 (45.2) | 0.095 |
| Diuretic | 3,582 (41.6) | 6,252 (54.0) | 0.249 |
| Metformin | 8,523 (99.1) | 10,659 (92.0) | 0.346 |
| Statin | 7,809 (90.8) | 9,922 (85.7) | 0.159 |
| Sulphonylurea | 5,500 (63.9) | 6,967 (60.2) | 0.078 |
| DPP-4 inhibitor | 5,758 (66.9) | 5,754 (49.7) | 0.355 |
| Insulin | 1,510 (17.6) | 3,052 (26.4) | 0.214 |

ACE = angiotensin-converting enzyme; ARB = angiotensin receptor blockers; BMI = body mass index; CVD = cardiovascular disease; DPP-4 = dipeptidyl peptidase 4; eGFR = estimated glomerular filtration rate; HbA1c = glycated haemoglobin; IMD = Index of Multiple Deprivation; IQR = interquartile range; NSAID = non-steroidal anti-inflammatory drug; Std. diff. = standardised difference; UACR = urine albumin to creatinine ratio

**Figure S6. Measure of balance across covariates between SGLT2i plus GLP-1 RA combination and GLP-1 RA monotherapy before (blue circles) and after (red circles) matching. Standardised differences <0.1 indicate good balance.**

**Figure S7.** **Frequency of SGLT2i plus GLP-1 RA combination users and matched GLP-1 RA monotherapy users, by duration of follow up (years).**

**Table S8.** **Effect of SGLT2i plus GLP-1 RA combination therapy on mean absolute change in eGFR (from baseline to 1- and 2-years follow-up) compared to GLP-1 RA monotherapy, stratified by UACR subgroup**

| **Outcome**  Exposure group | **No. with a measurement** | **Mean outcome** | **Crude Mean difference (95% CI)** | **Adjusted Mean difference (95% CI)†** |
| --- | --- | --- | --- | --- |
| **eGFR - 1 year (mL/min/1.73 m^2^)** | | | | |
| ***Subgroup: UACR <3 mg/mmol*** | | | | |
| SGLT2i + GLP-1 RA | 3,460 | -3.6 | 1.5 (0.72 to 2.19) | 1.7 (1.11 to 2.32) |
| GLP-1 RA | 3,451 | -5.1 | 0 (Reference) | 0 (Reference) |
| ***Subgroup: UACR ≥3 mg/mmol*** | | | | |
| SGLT2i + GLP-1 RA | 1,154 | -6.6 | 4.2 (2.74 to 5.72) | 3.4 (2.18 to 4.71) |
| GLP-1 RA | 1,005 | -10.9 | 0 (Reference) | 0 (Reference) |
| **eGFR - 2 years (mL/min/1.73 m^2^)** | | | | |
| ***Subgroup: UACR <3 mg/mmol*** | | | | |
| SGLT2i + GLP-1 RA | 2,498 | -4.1 | 1.6 (0.74 to 2.49) | 1.6 (0.84 to 2.31) |
| GLP-1 RA | 2,625 | -5.7 | 0 (Reference) | 0 (Reference) |
| ***Subgroup: UACR ≥3 mg/mmol*** | | | | |
| SGLT2i + GLP-1 RA | 805 | -8.5 | 3.4 (1.58 to 5.26) | 3.2 (1.57 to 4.74) |
| GLP-1 RA | 746 | -11.9 | 0 (Reference) | 0 (Reference) |

**†**Further adjusted for age, sex, ethnicity, socioeconomic status, body mass index (BMI), hypertension, cardiovascular disease, heart failure, haemorrhagic stroke, neuropathy, retinopathy, valvular heart disease, angiotensin-converting enzyme (ACE) inhibitors, angiotensin receptor blockers (ARB), diuretics, beta blockers, statins, acarbose, dipeptidyl peptidase-4 (DPP-4) inhibitors, insulin, metformin, sulphonylureas, thiazolidinediones, non-steroidal anti-inflammatory drugs (NSAID), duration of diabetes, HbA1c, eGFR, and UACR.

CI = confidence interval; eGFR = estimated glomerular filtration rate, HbA1c = glycated haemoglobin; UACR = urine albumin creatinine ratio

**Table S9.** **Effect of SGLT2i plus GLP-1 RA combination therapy on mean absolute change in eGFR (from baseline to 1- and 2-years follow-up) compared to GLP-1 RA monotherapy, stratified by HbA1c subgroup**

| **Outcome**  Exposure group | **No. with a measurement** | **Mean outcome** | **Crude Mean difference (95% CI)** | **Adjusted Mean difference (95% CI)†** |
| --- | --- | --- | --- | --- |
| **eGFR - 1 year (mL/min/1.73 m^2^)** | | | | |
| ***Subgroup: HbA1c <53 mmol/mol (<7%)*** | | | | |
| SGLT2i + GLP-1 RA | 340 | -4.6 | 2.7 (0.49 to 4.84) | 2.3 (0.52 to 4.18) |
| SGLT2i | 687 | -7.3 | 0 (Reference) | 0 (Reference) |
| ***Subgroup: HbA1c ≥53 and <64 mmol/mol (≥7 and <8%)*** | | | | |
| SGLT2i + GLP-1 RA | 802 | -3.9 | 3.0 (1.62 to 4.45) | 3.50 (2.33 to 4.66) |
| SGLT2i | 1293 | -6.9 | 0 (Reference) | 0 (Reference) |
| ***Subgroup: HbA1c ≥64 mmol/mol*** ***(≥8%)*** | | | | |
| SGLT2i + GLP-1 RA | 3472 | -4.5 | 1.4 (0.55 to 2.21) | 1.6 (0.97 to 2.30) |
| SGLT2i | 2476 | -5.8 | 0 (Reference) | 0 (Reference) |
| **eGFR - 2 years**  **(mL/min/1.73 m^2^)** | | | | |
| ***Subgroup: HbA1c <53 mmol/mol (<7%)*** | | | | |
| SGLT2i + GLP-1 RA | 212 | -7.4 | 1.1 (-1.73 to 3.91) | 1.1 (-1.28 to 3.53) |
| SGLT2i | 490 | -8.5 | 0 (Reference) | 0 (Reference) |
| ***Subgroup: HbA1c ≥53 and <64 mmol/mol (≥7 and <8%)*** | | | | |
| SGLT2i + GLP-1 RA | 590 | -3.7 | 3.9 (2.16 to 5.61) | 4.4 (2.98 to 5.82) |
| SGLT2i | 999 | -7.6 | 0 (Reference) | 0 (Reference) |
| ***Subgroup: HbA1c ≥64 mmol/mol*** | | | | |
| SGLT2i + GLP-1 RA | 2501 | -5.3 | 1.1 (0.12 to 2.09) | 1.1 (0.34 to 1.94) |
| SGLT2i | 1882 | -6.4 | 0 (Reference) | 0 (Reference) |

**†**Further adjusted for age, sex, ethnicity, socioeconomic status, body mass index (BMI), hypertension, cardiovascular disease, heart failure, haemorrhagic stroke, neuropathy, retinopathy, valvular heart disease, angiotensin-converting enzyme (ACE) inhibitors, angiotensin receptor blockers (ARB), diuretics, beta blockers, statins, acarbose, dipeptidyl peptidase-4 (DPP-4) inhibitors, insulin, metformin, sulphonylureas, thiazolidinediones, non-steroidal anti-inflammatory drugs (NSAID), duration of diabetes, HbA1c, eGFR, and UACR.

CI = confidence interval; eGFR = estimated glomerular filtration rate, HbA1c = glycated haemoglobin; UACR = urine albumin creatinine ratio

**Table S10.** **Effect of SGLT2i plus GLP-1 RA combination therapy on mean absolute change in eGFR (from baseline to 1- and 2-years follow-up) compared to GLP-1 RA monotherapy, stratified by previous use of RAS inhibitors**

| **Outcome**  Exposure group | **No. with a measurement** | **Mean outcome** | **Crude Mean difference (95% CI)** | **Adjusted Mean difference (95% CI)†** |
| --- | --- | --- | --- | --- |
| **eGFR - 1 year (mL/min/1.73 m^2^)** | | | | |
| ***Subgroup: RAS inhibitor*** | | | | |
| SGLT2i + GLP-1 RA | 3,143 | -5.5 | 2.4 (1.62 to 3.27) | 2.7 (1.96 to 3.34) |
| GLP-1 RA | 3,082 | -7.9 | 0 (Reference) | 0 (Reference) |
| ***Subgroup: No RAS inhibitor*** | | | | |
| SGLT2i + GLP-1 RA | 1,471 | -2.0 | 0.9 (-0.20 to 1.98) | 1.3 (0.38 to 2.12) |
| GLP-1 RA | 1,374 | -2.9 | 0 (Reference) | 0 (Reference) |
| **eGFR - 2 years (mL/min/1.73 m^2^)** | | | | |
| ***Subgroup: RAS inhibitor*** | | | | |
| SGLT2i + GLP-1 RA | 2,244 | -6.7 | 1.9 (0.88 to 2.88) | 2.3 (1.40 to 3.10) |
| GLP-1 RA | 2,334 | -8.5 | 0 (Reference) | 0 (Reference) |
| ***Subgroup: No RAS inhibitor*** | | | | |
| SGLT2i + GLP-1 RA | 1,059 | -1.9 | 1.8 (0.50 to 3.08) | 1.6 (0.56 to 2.68) |
| GLP-1 RA | 1,037 | -3.7 | 0 (Reference) | 0 (Reference) |

**†**Further adjusted for age, sex, ethnicity, socioeconomic status, body mass index (BMI), hypertension, cardiovascular disease, heart failure, haemorrhagic stroke, neuropathy, retinopathy, valvular heart disease, angiotensin-converting enzyme (ACE) inhibitors, angiotensin receptor blockers (ARB), diuretics, beta blockers, statins, acarbose, dipeptidyl peptidase-4 (DPP-4) inhibitors, insulin, metformin, sulphonylureas, thiazolidinediones, non-steroidal anti-inflammatory drugs (NSAID), duration of diabetes, HbA1c, eGFR, and UACR.

CI = confidence interval; eGFR = estimated glomerular filtration rate, HbA1c = glycated haemoglobin; UACR = urine albumin creatinine ratio

**Table S11.** **Effect of SGLT2i plus GLP-1 RA combination therapy on mean absolute change in eGFR (from baseline to 1- and 2-years follow-up) compared to GLP-1 RA monotherapy, including only individuals that were initiated therapies up to the 31^st^ December 2019**

| **Outcome**  Exposure group | **No. with a measurement** | **Mean outcome** | **Crude Mean difference (95% CI)** | **Adjusted Mean difference (95% CI)†** |
| --- | --- | --- | --- | --- |
| **eGFR - 1 year**  **(mL/min/1.73 m^2^)** | | | | |
| SGLT2i + GLP-1 RA | 2,399 | -3.4 | 1.4 (0.50 to 2.32) | 2.1 (1.35 to 2.83) |
| GLP-1 RA | 2,372 | -4.9 | 0 (Reference) | 0 (Reference) |
| **eGFR - 2 years**  **(mL/min/1.73 m^2^)** | | | | |
| SGLT2i + GLP-1 RA | 2,218 | -4.5 | 1.5 (0.50 to 2.49) | 2.0 (1.14 to 2.79) |
| GLP-1 RA | 2,191 | -6.0 | 0 (Reference) | 0 (Reference) |

**†**Further adjusted for age, sex, ethnicity, socioeconomic status, body mass index (BMI), hypertension, cardiovascular disease, heart failure, haemorrhagic stroke, neuropathy, retinopathy, valvular heart disease, angiotensin-converting enzyme (ACE) inhibitors, angiotensin receptor blockers (ARB), diuretics, beta blockers, statins, acarbose, dipeptidyl peptidase-4 (DPP-4) inhibitors, insulin, metformin, sulphonylureas, thiazolidinediones, non-steroidal anti-inflammatory drugs (NSAID), duration of diabetes, HbA1c, eGFR, and UACR.

CI = confidence interval; eGFR = estimated glomerular filtration rate, HbA1c = glycated haemoglobin; UACR = urine albumin creatinine ratio

**Table S12.** **Effect of SGLT2i plus GLP-1 RA combination therapy on mean absolute change in eGFR (from baseline to 1- and 2-years follow-up) compared to GLP-1 RA monotherapy, including all individuals in matching process, regardless of missing data**

| **Outcome**  Exposure group | **No. with a measurement** | **Mean outcome** | **Crude Mean difference (95% CI)** | **Adjusted Mean difference (95% CI)†** |
| --- | --- | --- | --- | --- |
| **eGFR - 1 year**  **(mL/min/1.73 m^2^)** | | | | |
| SGLT2i + GLP-1 RA | 5,189 | -4.1 | 1.8 (1.18 to 2.48) | 2.9 (2.42 to 3.47) |
| GLP-1 RA | 5,059 | -5.9 | 0 (Reference) | 0 (Reference) |
| **eGFR - 2 years**  **(mL/min/1.73 m^2^)** | | | | |
| SGLT2i + GLP-1 RA | 3,697 | -5.0 | 2.4 (1.61 to 3.19) | 3.3 (2.67 to 3.97) |
| GLP-1 RA | 3,775 | -7.3 | 0 (Reference) | 0 (Reference) |

**†**Further adjusted for age, sex, ethnicity, socioeconomic status, body mass index (BMI), hypertension, cardiovascular disease, heart failure, haemorrhagic stroke, neuropathy, retinopathy, valvular heart disease, angiotensin-converting enzyme (ACE) inhibitors, angiotensin receptor blockers (ARB), diuretics, beta blockers, statins, acarbose, dipeptidyl peptidase-4 (DPP-4) inhibitors, insulin, metformin, sulphonylureas, thiazolidinediones, non-steroidal anti-inflammatory drugs (NSAID), duration of diabetes, HbA1c, eGFR, and UACR.

CI = confidence interval; eGFR = estimated glomerular filtration rate, HbA1c = glycated haemoglobin; UACR = urine albumin creatinine ratio
