# Supplementary material for: Combining Sodium–Glucose Co‐Transporter‐2 Inhibitor Plus Glucagon‐Like Peptide‐1 Receptor Agonist for Glucose‐Lowering in Type 2 Diabetes: Effects of Drug Initiation Sequence on Kidney Function in Real‐World Clinical Practice (CombiKid Study)
Source: Diabetes Obes Metab. 2026 Jun 16;28(9):7950–63. doi: 10.1111/dom.70946 (PMC13448856; doi:10.1111/dom.70946)
Supplement: Supplementary file 2 [file DOM-28-7950-s002.docx]

# **Supplementary 1**

## Changes made to the study from the proposed methods of the study protocol

1. **Include individuals regardless of whether they have previously been prescribed a renin-angiotensin system (RAS) inhibitor (either an angiotensin-converting enzyme [ACE] inhibitor or an angiotensin receptor blocker [ARB]).**

In our study protocol (1), we reported that we would exclude individuals not previously prescribed a RAS inhibitor. This is because these medications are a recommended therapy for treating chronic kidney disease (CKD) (2, 3). However, the majority of the study population (~98%) had an estimated glomerular filtration rate (eGFR) at least 60 mL/min/1.73 m^2^, indicating that they did not have CKD at baseline. Before matching, more than 30% of individuals were not prescribed an ARB. Excluding people not prescribed RAS inhibitors would, therefore, restrict the sample size, and reduce generalisability of our study findings. This change was also important because it allowed us perform subgroup analysis according to whether previously prescribed a RAS inhibitor.

2. **Perform main analyses with complete cases only.**

We opted perform the main analyses with complete cases instead of multiple imputation to handle missing data; the latter method proved to be very computationally challenging. This was due to the design specification of time-based exposure sets, in which the same individual(s) could appear in multiple exposure sets. Moreover, for several outcome measures, the proportion of missing data was substantial (e.g. for urine ACR at 2 years, approximately 80% of individuals in the matched cohorts did not have a measurement); multiple imputation would become less reliable due there being limited observations in the data to accurately inform the imputation model (4).

For sensitivity analyses, we assigned any missing values to “Unknown” categories of affected variables before propensity score matching the SGLT2i plus GLP-1 RA combination users and monotherapy users (SGLT2i or GLP-1 RA only). This approach allowed us to match all individuals prescribed combination therapy with either users of SGLT2is or GLP-1 RAs only, regardless of any missing data across their baseline characteristics.

## R packages used for the statistical analyses

- base (5)
- ggplot2 (6)
- MatchIt (7)
- stats (5)
- tableone (8)

## References

1.	Feher M, Hinton W, Forbes A, Munro N, Joy M, Wheeler D, et al. Sodium-Glucose Cotransporter-2 Inhibitor and Glucagon-Like Peptide-1 Receptor Agonist Combination Therapy in Type 2 Diabetes: Protocol for a Kidney End Points Real-world Study (COMBi-KID Study). JMIR Res Protoc. 2022;11(7):e34206.

2.	de Boer IH, Khunti K, Sadusky T, Tuttle KR, Neumiller JJ, Rhee CM, et al. Diabetes Management in Chronic Kidney Disease: A Consensus Report by the American Diabetes Association (ADA) and Kidney Disease: Improving Global Outcomes (KDIGO). Diabetes Care. 2022;45(12):3075-90.

3.	National Insititute for Health Care and Excellence. Chronic kidney disease: assessment and management (NICE guideline NG203). 2021 [cited 2025 7th August].

4.	Sterne JA, White IR, Carlin JB, Spratt M, Royston P, Kenward MG, et al. Multiple imputation for missing data in epidemiological and clinical research: potential and pitfalls. Bmj. 2009;338:b2393.

5.	R Core Team. R: A language and environment for statistical computing. R Foundation for Statistical Computing, Vienna, Austria.2025 [cited 2025 23 October]. Available from: <https://www.R-project.org/>.

6.	Wickham H. ggplot2: Elegant Graphics for Data Analysis: Springer-Verlag New York; 2016.

7.	Ho DE, Imai, K., King, G., Stuart, E.A. MatchIt: Nonparametric Preprocessing for Parametric Causal Inference. Journal of Statistical Software. 2011;42(8):1-28.

8.	Yoshida K, Bartel, A. tableone: Create 'Table 1' to Describe Baseline Characteristics with or without Propensity Score Weights. 2020 [cited 2025 6 November]. Available from: <https://CRAN.R-project.org/package=tableone>.
